# Paracresotinic Acid

**Published:** 1890-06-14

**Authors:** 


### PARACRESOTINIC ACID.

Demme strongly recommends the use of paracresotinate of soda in intestinal derangements, especially in the acute gastrointestinal catarrh of young children. He advises the regulation of the doses according to age as follows in grammes :—

| Age in Years. | Maximum single dose. | Maximum daily quantity. |
|---|---|---|
| 2—4 | 0·1 — 0·25 | 0·5—1·0 |
| 5—10 | 0·25— 1·0 | 2·5—3·5 |
| 11—16 | 1·0 — 1·5 | 3·5—4·5 |

It should be given very cautiously to infants, beginning with doses not exceeding 0·01 gram, as in the formula.

| | | |
|---|---|---|
| Sod. paracresotine. | 0.1—0.2 | ..............grs iss—iii |
| Tr. Opii | Gutt. ii—iv | ..............♏ ii—iv |
| Cognac | 1·0 | ..............♏ xvi |
| Syrup | 5·0 | ..............ʒ iss |
| Aq. distill. | 25·0 | ..............ad ℥ iv. |

One teaspoonful every two hours.

